# Efficacy and safety of PD-1/PD-L1 inhibitors plus chemotherapy ± bevacizumab for recurrent or metastatic cervical cancer: a systematic review and meta-analysis of randomized controlled trials

**DOI:** 10.3389/fimmu.2026.1972995

**Published:** 2026-10-19

**Authors:** Raoshan Luo, Minghong Li, Li Ma, Zhilong Huang

**Affiliations:** 1 School of Clinical Medicine, Wuzhou Medical College, Wuzhou, China; 2 The First Affiliated Hospital of Guangxi University of Science and Technology, Guangxi University of Science and Technology, Liuzhou, China; 3 Guangzhou Women and Children’s Medical Center, Guangzhou Medical University, Guangzhou, China

**Keywords:** bevacizumab, cervical cancer, chemotherapy, clinical trials, meta-analysis, PD-1/PD-L1 inhibitors

## Abstract

**Background:**

The efficacy of adding PD-1/PD-L1 inhibitors to chemotherapy ± bevacizumab in recurrent or metastatic cervical cancer is under active investigation.

**Objective:**

To systematically evaluate the efficacy and safety of PD-1/PD-L1 inhibitors combined with chemotherapy ± bevacizumab in this setting through a meta-analysis of randomized controlled trials (RCTs).

**Methods:**

A systematic search of PubMed, Embase, Web of Science, Cochrane Library, Scopus, and ClinicalTrials.gov was conducted for RCTs published up to January 14, 2026. Primary outcomes were overall survival (OS) and progression-free survival (PFS); hazard ratios (HRs) were derived from reconstructed Kaplan-Meier curves.

**Results:**

Six RCTs involving 2,242 patients were included: 1,131 received PD-1/PD-L1 inhibitors plus chemotherapy ± bevacizumab, and 1,111 received chemotherapy ± bevacizumab alone. The combination regimen significantly prolonged median OS (28.7 vs. 19.4 months) and median PFS (12.0 vs. 8.7 months). Pooled risk ratios (RRs) for complete response, partial response, stable disease, progressive disease, and objective response rate were 1.606 (95% CI, 1.333–1.936), 1.127 (95% CI, 0.994–1.277), 0.733 (95% CI, 0.612–0.878), 0.460 (95% CI, 0.305–0.695), and 1.235 (95% CI, 1.153–1.322), respectively. The risk of any-grade adverse events did not differ significantly between groups (RR 0.999, 95% CI 0.991–1.008), whereas the combination was associated with an increased risk of grade ≥3 adverse events (RR 1.069, 95% CI 1.019–1.121). Frequently reported adverse reactions included nausea, anemia, constipation, arthralgia, neutropenia, hypertension, alopecia, and peripheral neuropathy.

**Conclusion:**

In patients with recurrent or metastatic cervical cancer, the addition of PD-1/PD-L1 inhibitors to chemotherapy ± bevacizumab significantly improves OS and PFS, though with a modest increase in high-grade toxicity.

**Systematic review registration:**

https://www.crd.york.ac.uk/PROSPERO/view/, identifier CRD420261472748

## Introduction

Cervical cancer remains one of the most common malignant tumors among women worldwide, ranking first in both incidence and mortality among gynecological malignancies, posing a severe threat to women’s health (1). High-risk human papillomavirus (HR-HPV) is the primary etiological agent, with approximately 91% of cervical cancer cases attributed to HPV infection (2). According to global epidemiological data from 2022, there were approximately 660,000 new cases and 350,000 deaths, highlighting a substantial disease burden (3). Although the incidence has declined in some regions due to the widespread implementation of screening programs and HPV vaccination, the prognosis for recurrent or metastatic cervical cancer (R/M CC) remains dismal, with a 5-year survival rate of only 10%–20% (4).

In the GOG-240 trial, platinum-based doublet chemotherapy combined with bevacizumab prolonged median overall survival to only 16.8 months for recurrent or metastatic cervical cancer. However, patients inevitably develop resistance, highlighting an urgent need for more effective novel therapeutic strategies (5). Recently, immune checkpoint inhibitors (ICIs), particularly PD-1/PD-L1 inhibitors, have emerged as a breakthrough in cervical cancer treatment. By alleviating T-cell immunosuppression and restoring anti-tumor immunity, they offer new hope for patients with R/M CC (6). Cervical cancer exhibits a high PD-L1 expression rate, rendering it relatively sensitive to immunotherapy (7). However, the objective response rate (ORR) of ICI monotherapy is approximately 17%; this limited efficacy suggests that immune activation alone is insufficient to overcome the complex immunosuppressive tumor microenvironment (8). Consequently, combination immunotherapy has become a major research focus, with the ‘triplet regimen’ combining ICIs, chemotherapy, and bevacizumab receiving particular attention (9). Mechanistically, chemotherapy not only exerts direct cytotoxic effects but also induces immunogenic cell death (ICD), promoting tumor antigen release and dendritic cell maturation, thereby enhancing anti-tumor immunity (10). Bevacizumab, a monoclonal antibody targeting vascular endothelial growth factor (VEGF), inhibits angiogenesis while promoting vascular normalization and alleviating tumor hypoxia, which facilitates T-cell infiltration and generates a synergistic effect with immunotherapy (11). Within the cervical cancer microenvironment, VEGF-mediated immunosuppression impedes dendritic cell maturation; anti-angiogenic therapy can reverse this process, thereby maximizing antitumor immune activation when combined with PD-1 inhibitors (12). Furthermore, the synergistic interaction between anti-angiogenic agents and ICIs in remodeling the tumor microenvironment has been extensively demonstrated, laying a solid foundation for clinical combination therapies (13).

Currently, based on pivotal trials such as KEYNOTE-826 and BEATCC, pembrolizumab or atezolizumab combined with chemotherapy (with or without bevacizumab) is recommended by multiple international guidelines as the first-line standard treatment for recurrent/metastatic cervical cancer (14). The final survival analysis from KEYNOTE-826 further solidified the long-term survival benefit of the pembrolizumab plus chemotherapy regimen (15). Despite these recommendations, whether bevacizumab is essential in combination immunotherapy, and the precise efficacy and safety differences between dual- and triple-agent regimens, remain significant clinical dilemmas (16). While the BEATCC study demonstrated substantial survival benefits with atezolizumab combined with bevacizumab and chemotherapy, the triple-agent regimen inevitably increased the risks of hypertension, proteinuria, and immune-related adverse events (17). Conversely, subgroup analyses from KEYNOTE-826 indicated that pembrolizumab plus chemotherapy confers survival benefits even in patients not receiving bevacizumab (18). This trade-off between efficacy and safety poses potential challenges to patient adherence and quality of life (13, 15). Furthermore, heterogeneity in enrollment criteria, PD-L1 testing thresholds, and regimens across existing trials makes it difficult to comprehensively assess the overall benefit-risk ratio based on single studies alone (19).

Although existing studies have explored immunotherapy combination regimens, and Wang et al. (20) published a network meta-analysis aiming to compare differences among various immunotherapy combination schemes, this study primarily relied on indirect comparisons. Furthermore, it does not fully encompass key update data from the BEATCC study published between 2023 and the present, as well as the final overall survival analysis from KEYNOTE-826. Therefore, this study conducts a meta-analysis to systematically evaluate the efficacy and safety of PD-1/PD-L1 inhibitor combinations with chemotherapy (with or without bevacizumab) in the treatment of recurrent or metastatic cervical cancer, aiming to provide the latest evidence-based guidance for clinical precision decision-making.

## Methods

### Study design

This meta-analysis was conducted in accordance with the PRISMA 2020 guidelines (21) and was prospectively registered with PROSPERO (CRD420261472748).

### Search strategy

We systematically searched PubMed, Embase, Web of Science, Cochrane Library, and Scopus for relevant studies published up to January 14, 2026. Ongoing or completed clinical trials were additionally identified through ClinicalTrials.gov. Search strategies were developed according to the PICOS framework and combined MeSH terms with free-text keywords, including “PD-1/PD-L1 inhibitors” and “cervical cancer.” Full search strings for each database are provided in the **Supplementary Materials**.

### Inclusion and exclusion criteria

The inclusion criteria were as follows: (1) Patients diagnosed with recurrent or metastatic cervical cancer; (2) Patients in the intervention group receiving PD-1/PD-L1 inhibitors combined with chemotherapy, with or without bevacizumab administration; (3) Patients in the control group receiving chemotherapy, with or without bevacizumab administration; (4) Studies reporting safety and efficacy outcomes, including adverse events, OS, PFS; (5) Study design: randomized controlled trials.

The exclusion criteria were as follows: (1) Non-clinical trial literature, such as case reports, conference abstracts, dissertations, letters, reviews, meta-analyses, editorials, registry records, retrospective studies, and animal studies; (2) Studies involving other cancers or diseases; (3) Irrelevant topics; (4) Studies lacking safety or efficacy data.

### Selection of articles

Duplicate records were identified and removed using EndNote 20 (Clarivate Analytics). Two reviewers independently screened titles and abstracts for relevance. During full-text assessment, **Supplementary Materials** were consulted when the main text lacked safety or efficacy data. For registered trials, ClinicalTrials.gov was searched by trial registration number (e.g., NCT number) to retrieve outcome data. Disagreements were resolved by consensus, with a third author arbitrating when necessary.

### Data extraction

Two reviewers independently extracted data using standardized predefined forms. Extracted information included: (1) study characteristics (e.g., region); (2) participant demographics and clinical features (e.g., sample size, age, ECOG performance status, FIGO stage, prior treatment history [e.g., prior surgery, radiotherapy, chemotherapy, concurrent chemoradiotherapy], treatment regimen, follow-up duration); and (3) safety and efficacy outcomes. Variables not reported in the original study were recorded as “not reported.” Discrepancies were resolved through discussion with a third investigator.

### Outcomes and definitions

The primary outcomes were overall survival (OS) and progression-free survival (PFS). OS was defined as the time from randomization to death from any cause. PFS was defined as the time from randomization to the first documented disease progression or death from any cause.

The secondary efficacy outcomes included objective response rate (ORR), complete response (CR), partial response (PR), stable disease (SD), and progressive disease (PD), all assessed according to the Response Evaluation Criteria in Solid Tumors (RECIST) version 1.1 (22). Briefly, CR was defined as the disappearance of all target and non-target lesions; PR as at least a 30% decrease in the sum of the diameters of target lesions relative to baseline; PD as at least a 20% increase in the sum of the diameters of target lesions relative to the nadir, or the appearance of new lesions; and SD as neither sufficient shrinkage to qualify for PR nor sufficient growth to qualify for PD. ORR was defined as the proportion of patients achieving CR or PR.

Safety endpoints included adverse events (AEs) of all grades, AEs of grade ≥3, and eight pre-specified clinically meaningful AEs (nausea, anemia, constipation, arthralgia, neutropenia, hypertension, alopecia, and peripheral neuropathy); all endpoints were expressed as the proportion of patients who experienced the respective event during treatment. AEs of all grades were defined as the proportion of patients who experienced at least one AE of any severity; AEs of grade ≥3 were defined as the proportion of patients who experienced at least one AE of grade 3 or higher.

### Quality assessment

Two independent reviewers evaluated the methodological quality of the included trials using the Cochrane RoB 2.0 tool for risk of bias assessment (23). Any discrepancies were resolved through discussion and consensus.

### Statistical analysis

Statistical analyses were performed using R version 4.4.2 and RStudio version 2026.05. Individual patient survival data were reconstructed from published Kaplan–Meier curves using the IPDfromKM tool, as described by Guyot et al. (24). For time-to-event outcomes (overall survival and progression-free survival), hazard ratios (HRs) and 95% confidence intervals (CIs) were estimated from Cox proportional hazards models fitted to the reconstructed individual patient data. Weighted mean differences (WMDs) with 95% CIs were used for continuous outcomes, and risk ratios (RRs) with 95% CIs for binary outcomes. The analysis unit for safety outcomes was defined as the proportion of patients who experienced at least one adverse event. The RR was calculated as the ratio of the proportion of patients experiencing adverse events in the treatment group to that in the control group. Heterogeneity was assessed using Cochran’s Q test and the I² statistic. Given the small number of included trials (n = 6), all effect estimates were pooled using a DerSimonian–Laird random-effects model with the Hartung–Knapp adjustment to provide more robust confidence intervals. P < 0.05 was considered statistically significant.

## Results

### Search results

**Figure 1** illustrates the literature screening process. A total of 7,523 records were identified, of which 2,418 duplicates were removed, leaving 5,105 records for title and abstract screening. After excluding ineligible records (e.g., conference abstracts, case reports, reviews, meta-analyses, animal experiments, registry data, correspondence, non-clinical trials, and studies not involving cervical cancer), 259 studies remained for further assessment. Of these, 217 non-randomized controlled trials were excluded, yielding 42 studies for full-text review. Finally, after full-text evaluation, **Supplementary Material** verification, and NCT number validation, 6 studies met the inclusion criteria.

**Figure 1:**
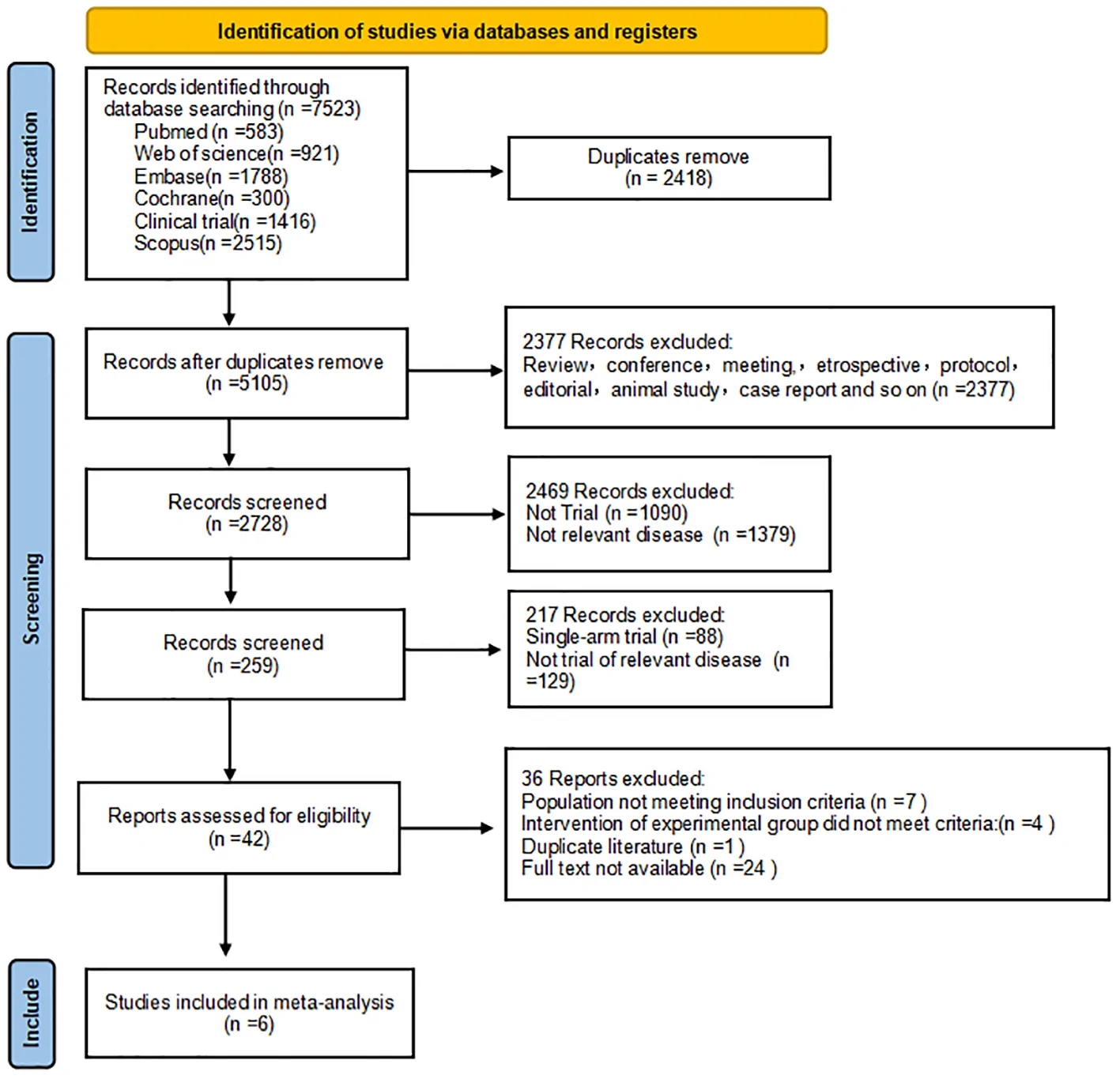
Flow chart of literature search strategies.

### Patient characteristics

This meta-analysis included six randomized controlled trials (RCTs) involving 2,242 patients, with 1,131 receiving PD-1/PD-L1 inhibitors plus chemotherapy (with or without bevacizumab) and 1,111 receiving chemotherapy (with or without bevacizumab). All participants had recurrent or metastatic cervical cancer. Except for one study that did not report median age, the median age was generally over 50 years. The PD-1/PD-L1 inhibitors investigated included cadonilimab, atezolizumab, and pembrolizumab. Baseline characteristics of the included trials and participants are presented in **Table 1**.

**Table 1:** Characteristics of the included studies.

Author year	Region	Sample size	Age (years)	ECOG performance status (I/C)	FIGO stage	Prior therapy (I/C)	Intervention	Control	Follow-up time(months,median)
Wu XH 2024	China	Total:445 I:222 C:223	NR	NR	NR	Total: CCRT:215 Bev:265	Cadonilimab 10mg/kg q3w + Platinum-based chemotherapy ± Bevacizumab 15 mg/kg	Placebo q3w + Platinum-based chemotherapy ± Bevacizumab 15 mg/kg	PFS:17.87 OS:25.63
Oaknin A 2024 (17)	Europe, Japan, United States	Total:410 I:206 C:204	I:51 C:52.5	0:138/128 1:68/73 None:0/3	I:31/42 II:61/53 III:14/12 IIIA:2/4 IIIB:34/22 IV:5/8 IVA:11/6 IVB:43/50	CCRT:70/85 Surgery:9/14 Surgery+CRT:64/44 Surgery+RT:5/11 RT:2/3	Atezolizumab 1200mg q3w + Cisplatin 50 mg/m² or Carboplatin AUC 5 + Paclitaxel 175 mg/m² + Bevacizumab 15 mg/kg	Cisplatin 50 mg/m² or Carboplatin AUC 5 + Paclitaxel 175 mg/m² + Bevacizumab 15mg/kg	32.9
Takekuma M 2025	Japan	Total:56 I:30 C:26	I:54.0 C:53.5	0:29/25 1:1/1	IVB:30/26	CCRT:12/12 Surgery:1/2 Surgery+CRT:4/3 Surgery+RT:1/0 RT:0/1	Atezolizumab 1200mg q3w + Cisplatin 50 mg/m² or Carboplatin AUC 5 + Paclitaxel 175 mg/m² + Bevacizumab 15 mg/kg	Cisplatin 50 mg/m² or Carboplatin AUC 5 + Paclitaxel 175 mg/m² + Bevacizumab 15mg/kg	33.6
Monk BJ 2023 (15)	Multinational(19 countries)	Total:617 I:308 C:309	I:51 C:50	0:178/170 1:128/139	I:67/58 II:85/93 III:5/8 IIIA:4/8 IIIB:46/42 IVA:7/4 IVB:94/96	Surgery:23/24 Surgery+CRT:49/56 Surgery+RT:22/23 CRT:125/118 RT:31/24	Pembrolizumab 200 mg every 3 weeks × 35 cycles + chemotherapy (cisplatin/carboplatin + paclitaxel) every 3 weeks × 6 cycles ± bevacizumab	Placebo q3w + Cisplatin 50 mg/m² or Carboplatin AUC 5 + Paclitaxel 175 mg/m² ± Bevacizumab 15 mg/kg	39.1
Tewari KS 2024 (18)	Multinational(19 countries)	Total:617 I:308 C:309	51	0:178/170 1:128/139	I:67/58 II:85/93 III:5/8 IIIA:4/8 IIIB:46/42 IVA:7/4 IVB:94/96	Bev:196/193 CRT:125/118	Pembrolizumab 200mg q3w + Cisplatin 50 mg/m² or Carboplatin AUC 5 + Paclitaxel 175 mg/m²± Bevacizumab 15 mg/kg	Placebo q3w + Cisplatin 50 mg/m² or Carboplatin AUC 5 + Paclitaxel 175 mg/m²± Bevacizumab 15 mg/kg	22
Kim YM 2025	East Asia	Total:97 I:57 C:40	I:56 C:52	0:45/25 1:12/15	I:15/9 II:17/8 IIIA:1/3 IIIB:5/2 IVB:19/18	Surgery:6/4 Surgery+CRT:10/5 Surgery+RT:3/1 CRT:27/16 RT:2/1	Pembrolizumab 200mg q3w×35 cycles + Cisplatin 50 mg/m² or Carboplatin AUC 5 + Paclitaxel 175 mg/m² ± Bevacizumab 15 mg/kg	Placebo q3w + Cisplatin 50 mg/m² or Carboplatin AUC 5 + Paclitaxel 175 mg/m² ± Bevacizumab 15 mg/kg	39.8

### Bias risk and evidence certainty

The methodological quality of the included trials was evaluated using the Cochrane RoB 2.0 tool, and all trials were judged to have a low risk of bias. The certainty of evidence was assessed using the GRADE PRO GDT framework. For the comparison between PD-1/PD-L1 inhibitor combined with chemotherapy (with or without bevacizumab) and chemotherapy (with or without bevacizumab), the certainty of evidence for overall survival (OS), progression-free survival (PFS), and adverse events (any grade and grade 3 or higher) was rated as moderate. The complete GRADE evidence profile is provided in the **Supplementary Material**.

### Efficacy

**Table 2** summarizes the efficacy outcomes. The risk ratios (RRs) for efficacy endpoints in the PD-1/PD-L1 inhibitor combined with chemotherapy (with or without bevacizumab) group, compared to the chemotherapy (with or without bevacizumab) group, were as follows: complete response (CR) (RR: 1.606; 95% CI: 1.333–1.936), partial response (PR) (RR: 1.127; 95% CI: 0.994–1.277), stable disease (SD) (RR: 0.733; 95% CI: 0.612–0.878), progressive disease (PD) (RR: 0.460; 95% CI: 0.305–0.695), and objective response rate (ORR) (RR: 1.235; 95% CI: 1.153–1.322). Detailed results are available in the **Supplementary Materials**.

**Table 2:** Summary of the forest plot results.

Outcomes	No. studies	No. patients	No. events	RR (95% CI)	Heterogeneity I2 (%)	Heterogeneity P-value
Objective response rate	5	1797	1162	1.235(1.153, 1.322)	24.2	0.2599
Complete response	5	1797	375	1.606(1.333, 1.936)	0	0.7308
Partial response	4	1387	573	1.127(0.994, 1.277)	0	0.8400
Stable disease	3	1331	361	0.733(0.612, 0.878)	29.5	0.2422
Progressive disease	3	1331	98	0.460(0.305, 0.695)	0	0.9547
Any AE	5	1789	1776	0.999(0.991, 1.008)	0	0.9974
Any grade ≥3 AE	5	1789	1418	1.069(1.019, 1.121)	16.4	0.3103
Nausea	5	1789	786	0.914(0.823, 1.015)	13.4	0.3287
Anemia	5	1789	947	1.128(1.034, 1.229)	0	0.8592
Constipation	5	1789	599	0.952(0.835, 1.084)	33.1	0.2006
Arthralgia	5	1789	475	1.164(0.997, 1.360)	27.5	0.2380
Neutropenia	5	1789	518	1.058(0.920, 1.217)	47.9	0.1040
Hypertension	5	1789	513	0.989(0.857, 1.143)	11.0	0.3435
Alopecia	5	1789	984	0.997(0.918, 1.083)	0	0.4931
Peripheral Neuropathy	5	1779	638	1.006(0.894, 1.131)	21.2	0.2795

### Safety

The risk ratio (RR) for any-grade adverse events (AEs) was 0.999 (95% CI: 0.991–1.008). For patients receiving PD-1/PD-L1 inhibitors combined with chemotherapy (with or without bevacizumab), the RR of grade 3 or higher AEs was 1.069 (95% CI: 1.019–1.121). The most common adverse reactions included nausea (RR: 0.914; 95% CI: 0.823–1.015), anemia (RR: 1.128; 95% CI: 1.034–1.229), constipation (RR: 0.952; 95% CI: 0.835–1.084), arthralgia (RR: 1.164; 95% CI: 0.997–1.360), neutropenia (RR: 1.058; 95% CI: 0.920–1.217), hypertension (RR: 0.989; 95% CI: 0.857–1.143), alopecia (RR: 0.997; 95% CI: 0.918–1.083), and peripheral neuropathy (RR: 1.006; 95% CI: 0.894–1.131). Detailed results are provided in the **Supplementary Materials**.

### Overall survival

**Figure 2** illustrates the overall survival (OS) of patients with recurrent or metastatic cervical cancer treated with PD-1/PD-L1 inhibitors combined with chemotherapy (with or without bevacizumab). Compared with the chemotherapy (with or without bevacizumab) group, the PD-1/PD-L1 inhibitor plus chemotherapy (with or without bevacizumab) regimen demonstrated significantly superior OS (HR = 0.65; 95% CI: 0.57–0.73; P < 0.0001). The median OS was 28.7 months in the immunotherapy combination group, compared to 19.4 months in the control group.

**Figure 2:**
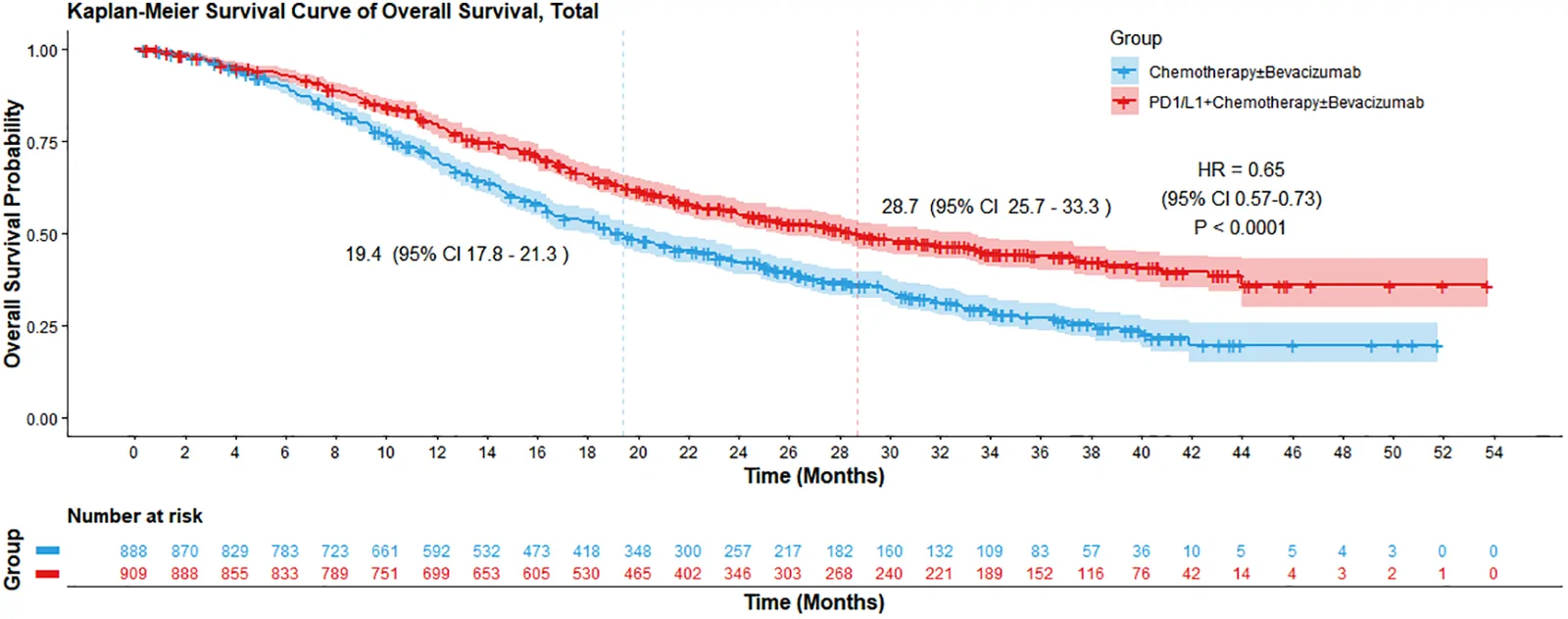
Kaplan-Meier curves for OS.

### Progression-free survival

**Figure 3** illustrates the progression-free survival (PFS) outcomes for patients with recurrent or metastatic cervical cancer treated with PD-1/PD-L1 inhibitors combined with chemotherapy (with or without bevacizumab). Compared with the chemotherapy (with or without bevacizumab) group, the PD-1/PD-L1 inhibitor plus chemotherapy (with or without bevacizumab) regimen demonstrated significantly superior PFS (HR = 0.62; 95% CI: 0.55–0.69; P < 0.0001). The median PFS was 12.0 months in the immunotherapy combination group, compared to 8.7 months in the control group.

**Figure 3:**
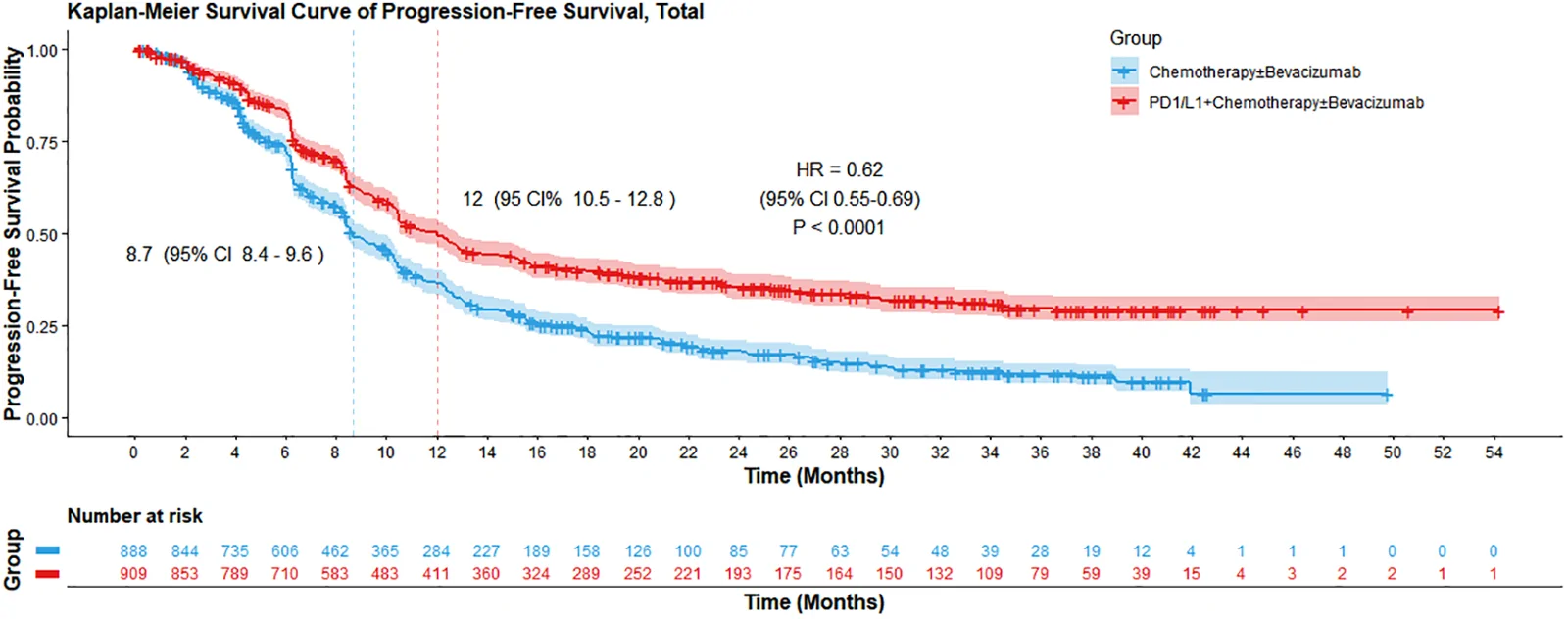
Kaplan-Meier curves for PFS.

### Sensitivity analysis and publication bias

To assess the robustness of the results, we conducted leave-one-out sensitivity analyses for all outcome measures. The findings demonstrated that the direction and statistical significance of the pooled effect sizes remained largely consistent upon the exclusion of individual studies, indicating that the results are stable.

Since this meta-analysis included only six studies, below the minimum threshold recommended for reliable funnel plot analysis (typically ≥ 10 studies), the statistical power of the funnel plot was limited. Consequently, the funnel plot was used solely as an exploratory visualization tool rather than a primary criterion for assessing publication bias. Nevertheless, funnel plots for most outcome measures displayed overall symmetric distributions, suggesting no clear evidence of publication bias. However, for specific outcomes such as stable disease (SD) and progressive disease (PD), interpretation remained impractical due to the extremely limited number of studies. Detailed results are provided in the **Supplementary Materials**.

## Discussion

This meta-analysis of six randomized controlled trials evaluated the efficacy of first-line PD-1/PD-L1 inhibitors plus chemotherapy ± bevacizumab in patients with recurrent or metastatic cervical cancer. The combination strategy was associated with statistically significant and clinically meaningful improvements in both primary endpoints. Specifically, the addition of immunotherapy reduced the risk of death by 35% (HR, 0.65; 95% CI, 0.57–0.73; P < 0.0001), translating into a 9.3-month extension of median overall survival (28.7 vs. 19.4 months). A similarly marked benefit was observed for progression-free survival, with a 38% reduction in the risk of progression or death (HR, 0.62; 95% CI, 0.55–0.69; P < 0.0001) and a 3.3-month prolongation of median PFS (12.0 vs. 8.7 months).

Irrespective of bevacizumab use, the PD-1/PD-L1 inhibitor–chemotherapy combination demonstrated superior overall antitumor activity compared with chemotherapy alone, while maintaining a generally manageable safety profile. The combination significantly increased the complete response rate (RR, 1.606; 95% CI, 1.333–1.936), suggesting that immunotherapy-based strategies may enable deeper tumor regression. The objective response rate was also significantly improved (RR, 1.235; 95% CI, 1.153–1.322), with low between-study heterogeneity (I² = 24.2%), underscoring the robustness of this finding. In parallel, the risks of progressive disease and stable disease were reduced by 54.0% (RR, 0.460) and 26.7% (RR, 0.733), respectively, lending further support to the enhanced disease control conferred by the combination. Although a trend toward a higher partial response rate was noted (RR, 1.127; 95% CI, 0.994–1.277), this difference did not achieve statistical significance, possibly owing to limited study power and event counts; validation in larger datasets is warranted.

With respect to safety, the overall incidence of adverse events (any grade) was virtually identical between groups (RR, 0.999; 95% CI, 0.991–1.008), indicating that the addition of immunotherapy did not materially increase the aggregate toxicity burden. The risks of common chemotherapy-associated toxicities—nausea (RR, 0.914; 95% CI, 0.823–1.015), constipation (RR, 0.952; 95% CI, 0.835–1.084), neutropenia (RR, 1.058; 95% CI, 0.920–1.217), hypertension (RR, 0.989; 95% CI, 0.857–1.143), alopecia (RR, 0.997; 95% CI, 0.918–1.083), and peripheral neuropathy (RR, 1.006; 95% CI, 0.894–1.131)—were comparable between arms, suggesting that the regimen does not exacerbate conventional gastrointestinal, hematological, or neuropathic effects. Notably, however, the risk of anemia was significantly elevated with the combination (RR, 1.128; 95% CI, 1.034–1.229), and arthralgia exhibited a nonsignificant trend toward higher incidence (RR, 1.164; 95% CI, 0.997–1.360). Grade ≥3 adverse events occurred at a modestly but significantly higher rate in the combination group (RR, 1.069; 95% CI, 1.019–1.121); such events are generally manageable in clinical practice with proactive supportive care. Heterogeneity across all safety endpoints was low (I² < 35%), reflecting consistent effect estimates.

The survival outcomes in this meta-analysis are highly consistent with those of previous reports (20, 25–27), all demonstrating that PD-1/PD-L1 inhibitors plus chemotherapy ± bevacizumab significantly prolong both OS and PFS. Before immune checkpoint inhibitors were introduced, first-line treatment for recurrent or metastatic cervical cancer relied primarily on platinum-based doublets such as paclitaxel plus carboplatin/cisplatin; even when bevacizumab was added, the overall prognosis remained poor (28). In the GOG 240 trial, median OS reached only 16.8 months in the bevacizumab–chemotherapy arm (5). Moreover, conventional chemotherapy has a limited capacity to mount durable adaptive immune responses or to establish immune memory, which contributes to a high risk of post-treatment relapse and persistently unfavorable long-term outcomes (29). The integration of immune checkpoint inhibitors with chemotherapy ± anti-angiogenic agents has now become the new first-line standard of care, fundamentally reshaping a treatment paradigm that was long dominated by platinum-based chemotherapy (15).

Our findings confirm that first-line immune checkpoint inhibitor–chemotherapy combinations ± bevacizumab are associated with significant improvements in OS and PFS over chemotherapy ± bevacizumab in recurrent or metastatic cervical cancer. Mechanistically, PD-1/PD-L1 inhibitors restore antitumor immunity by reversing T-cell exhaustion via checkpoint blockade (30). Adding bevacizumab to this backbone may further favorably modulate the tumor microenvironment through anti-angiogenic mechanisms. On one hand, vascular normalization induced by anti-VEGF therapy enhances tumor perfusion and facilitates intratumoral infiltration of CD8^+^ effector T cells; on the other, attenuation of VEGF signaling reduces the recruitment of myeloid-derived suppressor cells and regulatory T cells into the tumor microenvironment (31, 32). Subgroup analyses from the KEYNOTE-826 trial demonstrated consistent benefit from PD-1/PD-L1 inhibitors plus chemotherapy ± bevacizumab irrespective of PD-L1 expression level or histological subtype (18). Notably, even patients with squamous cell carcinoma or adenocarcinoma—tumor types historically regarded as having limited immunogenicity and low tumor mutational burden—obtained clinically meaningful improvements in OS and PFS (17, 18). These observations imply that the immunomodulatory effects of immunotherapy combined with anti-angiogenic therapy may create the prerequisites for immunotherapy benefit in these populations, while also informing future efforts to identify more precise predictive biomarkers (33, 34).

Several methodological strengths distinguish this study. First, the literature search was rigorous: a highly sensitive and specific strategy combining MeSH terms and keywords across PubMed, Embase, and the Cochrane Library was designed to capture all relevant RCTs. Second, the evidence base is both high-quality and contemporary. By restricting eligibility to RCTs, we pooled data from six eligible studies that represent the most recent evidence in this field, providing a solid foundation for the conclusions. Third, the outcome assessment was comprehensive and multidimensional. A structured framework was established that not only evaluated overall adverse events (any grade and grade ≥3) and eight specific clinically relevant toxicities, but also systematically examined efficacy endpoints including complete response, partial response, stable disease, progressive disease, and objective response rate, thereby fully capturing the antitumor activity and safety profile of the combination regimen. Fourth, data reconstruction was highly precise: using the IPDfromKM package to reconstruct individual patient data from published Kaplan–Meier curves substantially enhanced both the statistical power of the pooled survival analysis and the visual clarity of the results.

Several limitations should be considered. First, the included trials exhibited clinical and methodological heterogeneity. (i) The specific immune checkpoint inhibitors and combination regimens varied; they comprised PD-1 inhibitors (pembrolizumab, cadonilimab) and a PD-L1 inhibitor (atezolizumab), which differ in pharmacological properties, target affinity, and dosing schedules—all of which may influence efficacy and safety when combined with chemotherapy ± bevacizumab. (ii) The enrolled populations differed in geographic origin (Asia, Europe, Americas), ethnicity, HPV subtype distribution, PD-L1 testing platforms and cutoffs, and disease status (recurrent vs. metastatic). Patients with recurrent versus metastatic disease may represent clinically distinct populations in terms of disease burden, sites of disease, prior treatments, disease-free interval, and prognosis. Although some original studies performed regional or pathological subgroup analyses, such multidimensional baseline heterogeneity cannot be fully eliminated at the meta-analysis level and may introduce confounding interactions. (iii) Sample sizes ranged widely (56–617 patients), and follow-up durations and CTCAE versions (v4.0 vs. v5.0) varied across trials. Small studies are subject to greater random error, whereas larger studies may dominate the pooled estimates; shorter follow-up may miss immune-related delayed toxicities or late survival benefits, and differences in toxicity grading criteria may compromise cross-study comparability. Second, methods for assessing and reporting core endpoints were not fully standardized. While most trials reported PFS, OS, and adverse events, some assessed PFS by investigator review rather than by blinded independent central review, which may introduce assessment bias in open-label designs. In addition, certain exploratory subgroup analyses were not corrected for multiplicity. Third, formal evaluation of publication bias was constrained by the small number of included studies (n = 6); consequently, funnel plots were included in the **Supplementary Materials** for exploratory purposes only, as statistical power to distinguish true asymmetry from chance or heterogeneity is limited. Fourth, the modest overall sample size and limited available data precluded meaningful subgroup analyses. Specifically, we were unable to perform subgroup analyses based on HPV status, bevacizumab use, or key baseline demographic and clinical characteristics (e.g., age, race, ECOG performance status, PD-1/PD-L1 inhibitor type, PD-L1 status, and prior treatment history), as well as disease status (recurrent vs. metastatic). HPV status is a biologically important factor that may influence tumor characteristics and treatment responses in cervical cancer. However, because this meta-analysis primarily evaluated available aggregate data and we lacked access to individual patient data (IPD), we were unable to accurately classify patients and perform stratified analyses based on HPV status. This was primarily attributed to the lack of access to IPD and incomplete reporting of these variables across the included trials. Consequently, the specific additive benefit of bevacizumab within PD-1/PD-L1-based combination regimens, as well as its heterogeneous impact on safety, could not be precisely assessed. These limitations highlight the urgent need for larger, well-designed randomized controlled trials and IPD-based meta-analyses to further validate and refine the present findings and to further explore HPV status as a potential effect modifier.

Evaluating the clinical benefit of combining PD-1/PD-L1 inhibitors with chemotherapy (± bevacizumab) in the first-line setting is crucial for optimizing treatment decisions in recurrent or metastatic cervical cancer. At 12 months, the progression-free survival (PFS) rate was 49.8% in the combination therapy group versus 36.4% in the control group. This represents an absolute risk reduction (ARR) of 13.4% and a corresponding number needed to treat (NNT) of 8, indicating that treating 8 additional patients yields one additional patient achieving 12-month PFS. Similarly, the 12-month overall survival (OS) rates were 79.6% and 70.6%, respectively, resulting in an ARR of 9.0% and an NNT of 11. This means 11 additional patients must be treated to yield one additional patient achieving 12-month OS. Collectively, these NNT values demonstrate that the combination therapy confers a clear survival benefit in this patient population, with particularly pronounced efficacy in delaying disease progression.

In terms of safety, combination therapy increases the absolute risk of grade ≥3 adverse events by 5.6% (NNH = 18) and anemia by 6.4% (NNH = 16). These findings suggest that close monitoring for severe toxicities and the implementation of preventive interventions are warranted in clinical practice to optimize the risk–benefit balance. Comparing the NNT and NNH values reveals that the therapeutic benefit (NNT = 8 for PFS; NNT = 11 for OS) and the safety cost (NNH = 16–18) are of a similar magnitude. This suggests that although the regimen confers a statistically significant and clinically meaningful survival improvement in the overall population, its benefit–risk ratio remains relatively limited; a considerable proportion of patients may experience toxicity without achieving substantial survival prolongation.

Notably, these NNT and NNH estimates are derived from the overall population. Due to the limited number of included studies and the heterogeneity in PD-L1 detection platforms and positivity thresholds across trials, this meta-analysis could not perform stratified NNT calculations based on PD-L1 expression status. Consequently, the precise quantification of benefit–risk differences among PD-L1 subgroups requires further investigation using individual patient data. Additionally, the added value of bevacizumab in the combination regimen remains unclear. While a three-drug regimen incorporating bevacizumab can confer survival benefits, anti-angiogenic agents may inherently increase the risk of specific adverse events, such as hypertension and proteinuria. Therefore, the risk–benefit profile should be carefully weighed on an individual basis during clinical decision-making.

Future randomized trials should prioritize several areas to strengthen the evidence base. First and foremost, factorial designs or prespecified bevacizumab-stratified randomization are urgently warranted in PD-1/PD-L1 inhibitor-based combination regimens to evaluate the actual incremental benefit of adding bevacizumab to chemotherapy plus immunotherapy. This incremental benefit should be carefully weighed against the potential additional toxicities and substantial costs associated with bevacizumab, so as to better inform risk–benefit and cost-effectiveness assessments. This issue remained unresolved in the current meta-analysis owing to the limited number of included studies. Second, standardizing PD-L1 testing platforms, cutoffs, and histological subtype definitions across trials would reduce inter-study heterogeneity and facilitate more precise patient enrichment strategies in the immuno-oncology era. Third, future studies should adopt blinded independent central review for PFS assessment as the standard and incorporate mature overall survival data with extended follow-up to fully capture long-term outcomes and late-onset immune-related toxicities. Fourth, the integration of biomarker-driven stratified designs—incorporating HPV genotype, tumor mutational burden, and immune microenvironment profiling—may help identify subgroups most likely to derive durable benefit. Finally, large-scale, international multicenter RCTs with harmonized safety reporting using the latest CTCAE criteria are essential to refine the risk–benefit assessment of these combination regimens and to guide individualized treatment decisions in recurrent or metastatic cervical cancer.

## Conclusion

In conclusion, PD-1/PD-L1 inhibitors plus chemotherapy ± bevacizumab significantly improve overall survival and progression-free survival in patients with recurrent or metastatic cervical cancer. This survival benefit is accompanied by a modest increase in the incidence of grade ≥3 adverse events, underscoring the need for rigorous toxicity monitoring, graded management, and preventive interventions to optimize the risk–benefit balance in clinical practice. Given the limitations of this meta-analysis, future high-quality randomized trials with standardized designs, larger sample sizes, extended follow-up, and prespecified subgroup analyses are warranted to validate these findings and to clarify the specific additive value of bevacizumab within this combination.

## Data Availability

The original contributions presented in the study are included in the article/**Supplementary Material**. Further inquiries can be directed to the corresponding author/s.
