# Supplementary material for: Efficacy and safety of PD-1/PD-L1 inhibitors plus chemotherapy ± bevacizumab for recurrent or metastatic cervical cancer: a systematic review and meta-analysis of randomized controlled trials
Source: Front Immunol. 2026 Oct 19;17:1972995. doi: 10.3389/fimmu.2026.1972995 (PMC13724596; doi:10.3389/fimmu.2026.1972995)
Supplement: Supplementary file 1 [file Table1.docx]

**Methods:** Search strategy

**eFigure 1.** Summary of risks of bias of all included studies

**eFigure 2.** The GRADE Rating for the comparisons of PD-1/PD-L1 + chemotherapy ±bevacizumab vs chemotherapy±bevacizumab in all included studies

**eFigure 3.** Forest plot of Complete Response Rate

**eFigure 4.** Forest plot of Objective Response Rate

**eFigure 5.** Forest plot of Progressive Disease Rate

**eFigure 6.** Forest plot of Partial Response Rate

**eFigure 7.** Forest plot of Stable desease Rate

**eFigure 8.** Forest plot of Any AE

**eFigure 9.** Forest plot of Any grade ≥3 AE

**eFigure 10.** Forest plot of Nausea

**eFigure 11.** Forest plot of Anemia

**eFigure 12.** Forest plot of Constipation

**eFigure 13.** Forest plot of Arthralgia

**eFigure 14.** Forest plot of Neutropenia

**eFigure 15.** Forest plot of Hypertension

**eFigure 16.** Forest plot of Alopecia

**eFigure 17.** Forest plot of Peripheral Neuropathy

**eFigure 18.** Sensitivity analysis of Complete Response Rate

**eFigure 19.** Sensitivity analysis of Objective Response Rate

**eFigure 20.** Sensitivity analysis of Progressive Disease Rate

**eFigure 21.** Sensitivity analysis of Partial Response Rate

**eFigure 22.** Sensitivity analysis of Stable desease Rate

**eFigure 23.** Sensitivity analysis of Any AE

**eFigure 24.** Sensitivity analysis of Any grade ≥3 AE

**eFigure 25.** Sensitivity analysis of Nausea

**eFigure 26.** Sensitivity analysis of Anemia

**eFigure 27.** Sensitivity analysis of Constipation

**eFigure 28.** Sensitivity analysis of Arthralgia

**eFigure 29.** Sensitivity analysis of Neutropenia

**eFigure 30.** Sensitivity analysis of Hypertension

**eFigure 31.** Sensitivity analysis of Alopecia

**eFigure 32.** Sensitivity analysis of Peripheral Neuropathy

**eFigure 33.** Funnel plot of Complete Response Rate

**eFigure 34.** Funnel plot of Objective Response Rate

**eFigure 35.** Funnel plot of Progressive Disease Rate

**eFigure 36.** Funnel plot of Partial Response Rate

**eFigure 37.** Funnel plot of Stable desease Rate

**eFigure 38.** Funnel plot of Any AE

**eFigure 39.** Funnel plot of Any grade ≥3 AE

**eFigure 40.** Funnel plot of Nausea

**eFigure 41.** Funnel plot of Anemia

**eFigure 42.** Funnel plot of Constipation

**eFigure 43.** Funnel plot of Arthralgia

**eFigure 44.** Funnel plot of Neutropenia

**eFigure 45.** Funnel plot of Hypertension

**eFigure 46.** Funnel plot of Alopecia

**eFigure 47.** Funnel plot of Peripheral Neuropathy

**eFigure 48.** Forest plot of PFS

**eFigure 49.** Sensitivity analysis of PFS

**eFigure 50.** Funnel plot of PFS

**eFigure 51.** Forest plot of OS

**eFigure 52.** Sensitivity analysis of OS

**eFigure 53.** Funnel plot of OS

ClinicalTrials.gov

Search Details: Viewing 1-10 out of 1,416 studies for: cervical OR Cervix | Ipilimumab OR MDX010 OR Yervoy OR Tremelimumab OR ticilimumab OR CP675206 OR Cadonilimab OR Epaloprilotovirilimab OR Pembrolizumab OR Keytruda OR lambrolizumab OR Nivolumab OR Opdivo OR Toripalimab OR Tislelizumab OR tevimbra OR Camrelizumab OR carrelizumab OR Cemiplimab OR REGN2810 OR Sintilimab OR IBI308 OR Zimberelimab OR Prolgolimab OR Dostarlimab OR Jemperli OR GSK4057190 OR Penpulimab OR Serplulimab OR Potelimab OR Atezolizumab OR Tecentriq OR RG7446 OR MPDL3280A OR Durvalumab OR Imfinzi OR MEDI4736 OR Avelumab OR Bavencio OR MSB0010718C OR MSB0010682 OR Envafolimab OR KN035 OR Sugemalimab OR Sokazolimab OR Tagolimumab OR Adebrelimab 1416

| PubMed | | |
| --- | --- | --- |
| No. | Query | Results |
| #1 | (cervical[Title/Abstract]) OR (Cervix[Title/Abstract]) | 325138 |
| #2 | ((((((((((((((((((((((((((((((((((((((((((((((Ipilimumab[Title/Abstract]) OR (MDX010[Title/Abstract])) OR (Yervoy[Title/Abstract])) OR (Tremelimumab[Title/Abstract])) OR (ticilimumab[Title/Abstract])) OR (CP675206[Title/Abstract])) OR (Cadonilimab[Title/Abstract])) OR (Epaloprilotovirilimab[Title/Abstract])) OR (Pembrolizumab[Title/Abstract])) OR (Keytruda[Title/Abstract])) OR (lambrolizumab[Title/Abstract])) OR (Nivolumab[Title/Abstract])) OR (Opdivo[Title/Abstract])) OR (Toripalimab[Title/Abstract])) OR (Tislelizumab[Title/Abstract])) OR (tevimbra[Title/Abstract])) OR (Camrelizumab[Title/Abstract])) OR (carrelizumab[Title/Abstract])) OR (Cemiplimab[Title/Abstract])) OR (REGN2810[Title/Abstract])) OR (Sintilimab[Title/Abstract])) OR (IBI308[Title/Abstract])) OR (Zimberelimab[Title/Abstract])) OR (Prolgolimab[Title/Abstract])) OR (Dostarlimab[Title/Abstract])) OR (Jemperli[Title/Abstract])) OR (GSK4057190[Title/Abstract])) OR (Penpulimab[Title/Abstract])) OR (Serplulimab[Title/Abstract])) OR (Potelimab[Title/Abstract])) OR (Atezolizumab[Title/Abstract])) OR (Tecentriq[Title/Abstract])) OR (RG7446[Title/Abstract])) OR (MPDL3280A[Title/Abstract])) OR (Durvalumab[Title/Abstract])) OR (Imfinzi[Title/Abstract])) OR (MEDI4736[Title/Abstract])) OR (Avelumab[Title/Abstract])) OR (Bavencio[Title/Abstract])) OR (MSB0010718C[Title/Abstract])) OR (MSB0010682[Title/Abstract])) OR (Envafolimab[Title/Abstract])) OR (KN035[Title/Abstract])) OR (Sugemalimab[Title/Abstract])) OR (Sokazolimab[Title/Abstract])) OR (Tagolimumab[Title/Abstract])) OR (Adebrelimab[Title/Abstract]) | 31795 |
| #3 | #1 AND #2 | 583 |

| Cochrane library | | |
| --- | --- | --- |
| No. | Query | Results |
| #1 | (cervical OR Cervix):ti,ab,kw | 32442 |
| #2 | (Ipilimumab OR MDX010 OR Yervoy OR Tremelimumab OR ticilimumab OR CP675206 OR Cadonilimab OR Epaloprilotovirilimab OR Pembrolizumab OR Keytruda OR lambrolizumab OR Nivolumab OR Opdivo OR Toripalimab OR Tislelizumab OR tevimbra OR Camrelizumab OR carrelizumab OR Cemiplimab OR REGN2810 OR Sintilimab OR IBI308 OR Zimberelimab OR Prolgolimab OR Dostarlimab OR Jemperli OR GSK4057190 OR Penpulimab OR Serplulimab OR Potelimab OR Atezolizumab OR Tecentriq OR RG7446 OR MPDL3280A OR Durvalumab OR Imfinzi OR MEDI4736 OR Avelumab OR Bavencio OR MSB0010718C OR MSB0010682 OR Envafolimab OR KN035 OR Sugemalimab OR Sokazolimab OR Tagolimumab OR Adebrelimab):ti,ab,kw | 13505 |
| #3 | #1 AND #2 | 300 |

| Embase | | |
| --- | --- | --- |
| No. | Query | Results |
| #1 | cervical:ab,ti,kw OR Cervix:ab,ti,kw | 449800 |
| #2 | Ipilimumab:ab,ti,kw OR MDX010:ab,ti,kw OR Yervoy:ab,ti,kw OR Tremelimumab:ab,ti,kw OR ticilimumab:ab,ti,kw OR CP675206:ab,ti,kw OR Cadonilimab:ab,ti,kw OR Epaloprilotovirilimab:ab,ti,kw OR Pembrolizumab:ab,ti,kw OR Keytruda:ab,ti,kw OR lambrolizumab:ab,ti,kw OR Nivolumab:ab,ti,kw OR Opdivo:ab,ti,kw OR Toripalimab:ab,ti,kw OR Tislelizumab:ab,ti,kw OR tevimbra:ab,ti,kw OR Camrelizumab:ab,ti,kw OR carrelizumab:ab,ti,kw OR Cemiplimab:ab,ti,kw OR REGN2810:ab,ti,kw OR Sintilimab:ab,ti,kw OR IBI308:ab,ti,kw OR Zimberelimab:ab,ti,kw OR Prolgolimab:ab,ti,kw OR Dostarlimab:ab,ti,kw OR Jemperli:ab,ti,kw OR GSK4057190:ab,ti,kw OR Penpulimab:ab,ti,kw OR Serplulimab:ab,ti,kw OR Potelimab:ab,ti,kw OR Atezolizumab:ab,ti,kw OR Tecentriq:ab,ti,kw OR RG7446:ab,ti,kw OR MPDL3280A:ab,ti,kw OR Durvalumab:ab,ti,kw OR Imfinzi:ab,ti,kw OR MEDI4736:ab,ti,kw OR Avelumab:ab,ti,kw OR Bavencio:ab,ti,kw OR MSB0010718C:ab,ti,kw OR MSB0010682:ab,ti,kw OR Envafolimab:ab,ti,kw OR KN035:ab,ti,kw OR Sugemalimab:ab,ti,kw OR Sokazolimab:ab,ti,kw OR Tagolimumab:ab,ti,kw OR Adebrelimab:ab,ti,kw | 79837 |
| #3 | #1 AND #2 | 1788 |

| Web of science | | |
| --- | --- | --- |
| No. | Query | Results |
| #1 | TS=(cervical OR Cervix) | 204306 |
| #2 | TS=(Ipilimumab OR MDX010 OR Yervoy OR Tremelimumab OR ticilimumab OR CP675206 OR Cadonilimab OR Epaloprilotovirilimab OR Pembrolizumab OR Keytruda OR lambrolizumab OR Nivolumab OR Opdivo OR Toripalimab OR Tislelizumab OR tevimbra OR Camrelizumab OR carrelizumab OR Cemiplimab OR REGN2810 OR Sintilimab OR IBI308 OR Zimberelimab OR Prolgolimab OR Dostarlimab OR Jemperli OR GSK4057190 OR Penpulimab OR Serplulimab OR Potelimab OR Atezolizumab OR Tecentriq OR RG7446 OR MPDL3280A OR Durvalumab OR Imfinzi OR MEDI4736 OR Avelumab OR Bavencio OR MSB0010718C OR MSB0010682 OR Envafolimab OR KN035 OR Sugemalimab OR Sokazolimab OR Tagolimumab OR Adebrelimab) | 62579 |
| #3 | #1 AND #2 | 921 |

| Scopus | | |
| --- | --- | --- |
| No. | Query | Results |
| #1 | TITLE-ABS-KEY(cervical OR Cervix) | 494167 |
| #2 | TITLE-ABS-KEY(Ipilimumab OR MDX010 OR Yervoy OR Tremelimumab OR ticilimumab OR CP675206 OR Cadonilimab OR Epaloprilotovirilimab OR Pembrolizumab OR Keytruda OR lambrolizumab OR Nivolumab OR Opdivo OR Toripalimab OR Tislelizumab OR tevimbra OR Camrelizumab OR carrelizumab OR Cemiplimab OR REGN2810 OR Sintilimab OR IBI308 OR Zimberelimab OR Prolgolimab OR Dostarlimab OR Jemperli OR GSK4057190 OR Penpulimab OR Serplulimab OR Potelimab OR Atezolizumab OR Tecentriq OR RG7446 OR MPDL3280A OR Durvalumab OR Imfinzi OR MEDI4736 OR Avelumab OR Bavencio OR MSB0010718C OR MSB0010682 OR Envafolimab OR KN035 OR Sugemalimab OR Sokazolimab OR Tagolimumab OR Adebrelimab) | 76812 |
| #3 | #1 AND #2 | 2515 |

**eFigure 1.** Summary of risks of bias of all included studies

**eFigure 2.** The GRADE Rating for the comparisons of PD-1/PD-L1 + chemotherapy ±bevacizumab vs chemotherapy±bevacizumab in all included studies

**eFigure 3.** Forest plot of Complete Response Rate

**eFigure 4.** Forest plot of Objective Response Rate

**eFigure 5.** Forest plot of Progressive Disease Rate

**eFigure 6.** Forest plot of Partial Response Rate

**eFigure 7.** Forest plot of Stable desease Rate

**eFigure 8.** Forest plot of Any AE

**eFigure 9.** Forest plot of Any grade ≥3 AE

**eFigure 10.** Forest plot of Nausea

**eFigure 11.** Forest plot of Anemia

**eFigure 12.** Forest plot of Constipation

**eFigure 13.** Forest plot of Arthralgia

**eFigure 14.** Forest plot of Neutropenia

**eFigure 15.** Forest plot of Hypertension

**eFigure 16.** Forest plot of Alopecia

**eFigure 17.** Forest plot of Peripheral Neuropathy

**eFigure 18.** Sensitivity analysis of Complete Response Rate

**eFigure 19.** Sensitivity analysis of Objective Response Rate

**eFigure 20.** Sensitivity analysis of Progressive Disease Rate

**eFigure 21.** Sensitivity analysis of Partial Response Rate

**eFigure 22.** Sensitivity analysis of Stable desease Rate

**eFigure 23.** Sensitivity analysis of Any AE

**eFigure 24.** Sensitivity analysis of Any grade ≥3 AE

**eFigure 25.** Sensitivity analysis of Nausea

**eFigure 26.** Sensitivity analysis of Anemia

**eFigure 27.** Sensitivity analysis of Constipation

**eFigure 28.** Sensitivity analysis of Arthralgia

**eFigure 29.** Sensitivity analysis of Neutropenia

**eFigure 30.** Sensitivity analysis of Hypertension

**eFigure 31.** Sensitivity analysis of Alopecia

**eFigure 32.** Sensitivity analysis of Peripheral Neuropathy

**eFigure 33.** Funnel plot of Complete Response Rate

**eFigure 34.** Funnel plot of Objective Response Rate

**eFigure 35.** Funnel plot of Progressive Disease Rate

**eFigure 36.** Funnel plot of Partial Response Rate

**eFigure 37.** Funnel plot of Stable desease Rate

**eFigure 38.** Funnel plot of Any AE

**eFigure 39.** Funnel plot of Any grade ≥3 AE

**eFigure 40.** Funnel plot of Nausea

**eFigure 41.** Funnel plot of Anemia

**eFigure 42.** Funnel plot of Constipation

**eFigure 43.** Funnel plot of Arthralgia

**eFigure 44.** Funnel plot of Neutropenia

**eFigure 45.** Funnel plot of Hypertension

**eFigure 46.** Funnel plot of Alopecia

**eFigure 47.** Funnel plot of Peripheral Neuropathy

**eFigure 48.** Forest plot of PFS

**eFigure 49.** Sensitivity analysis of PFS

**eFigure 50.** Funnel plot of PFS

**eFigure 51.** Forest plot of OS

**eFigure 52.** Sensitivity analysis of OS

**eFigure 53.** Funnel plot of OS
